# Role of SIRT1 in Potentially Toxic Trace Elements (Lead, Fluoride, Aluminum and Cadmium) Associated Neurodevelopmental Toxicity

**DOI:** 10.1007/s12011-024-04116-5

**Published:** 2024-02-28

**Authors:** Aqsa Fathima, Newly Bagang, Nitesh Kumar, Somasish Ghosh Dastidar, Smita Shenoy

**Affiliations:** 1 Department of Pharmacology, Kasturba Medical College, Manipal, Manipal Academy of Higher Education, Manipal, Karnataka 576104 India; 2 Department of Pharmacology and Toxicology, National Institute of Pharmaceutical Education and Research (NIPER), Hajipur, Industrial area Hajipur, Vaishali, Bihar 844102 India; 3 Centre for Molecular Neurosciences, Kasturba Medical College, Manipal, Manipal Academy of Higher Education, Manipal, Karnataka 576104 India

**Keywords:** SIRT1, Neurotoxicity, Mitochondrial dysfunction, Memory, Neurogenesis

## Abstract

The formation of the central nervous system is a meticulously planned and intricate process. Any modification to this process has the potential to disrupt the structure and operation of the brain, which could result in deficiencies in neurological growth. When neurotoxic substances are present during the early stages of development, they can be exceptionally dangerous. Prenatally, the immature brain is extremely vulnerable and is therefore at high risk in pregnant women associated with occupational exposures. Lead, fluoride, aluminum, and cadmium are examples of possibly toxic trace elements that have been identified as an environmental concern in the aetiology of a number of neurological and neurodegenerative illnesses. SIRT1, a member of the sirtuin family has received most attention for its potential neuroprotective properties. SIRT1 is an intriguing therapeutic target since it demonstrates important functions to increase neurogenesis and cellular lifespan by modulating multiple pathways. It promotes axonal extension, neurite growth, and dendritic branching during the development of neurons. Additionally, it contributes to neurogenesis, synaptic plasticity, memory development, and neuroprotection. This review summarizes the possible role of SIRT1 signalling pathway in potentially toxic trace elements -induced neurodevelopmental toxicity, highlighting some molecular pathways such as mitochondrial biogenesis, CREB/BDNF and PGC-1α/NRF1/TFAM.

## Introduction

The process of brain development is incredibly complicated, and its interruption will have long-term effects both structurally and functionally in the brain, including neurodevelopmental disorders (NDDs). While many disorders underlying NDDs are genetically based, environmental factors can also affect the development of such disorders. More than 200 industrially produced chemicals have been linked to neurodevelopmental toxicity in humans, and exposure to these chemicals through consumables or environmental pollution is a serious health risk, especially for children [1, 2]. Among the environmental factors, trace elements can pose a detrimental effect on neurodevelopment. According to WHO, trace elements are classified into 3 categories: essential trace elements, probably essential elements and potentially toxic trace elements [3]. Potentially toxic trace elements (PTEs) are chemical components that are naturally found in the environment. It is highly likely that PTEs are able to accumulate in plants and animals, where they are highly persistent in nature, and then enter the human body [4–6]. These toxic elements have been released into the environment as an outcome of natural events and technological applications, increasing concerns about the potential effects on human health and the ecosystem. Even at lower exposure levels, they are known to cause damage to various organs [7]. The developing brain is a highly vulnerable target organ and increased exposure to trace elements may have negative effects on human health [8] This review is focused on neurodevelopmental effects of potentially toxic trace elements – aluminum, cadmium, lead and fluoride. Clinical evidence for potentially toxic trace elements-based neurotoxicity is summarised in Table 1. Fluoride (Fl), lead (Pb), cadmium (Cd), and aluminum (Al) are commonly known toxic trace elements that people are exposed to, in general. These affect the liver, kidney, and brain, causing nephrotoxicity, hepatotoxicity, and neurotoxicity, respectively [9]. As the central nervous system is highly susceptibile, early exposure to these substances raises concerns [10]. Low-dose PTEs exposure in non-occupational settings could pose a substantial risk, particularly to unborn babies and young children [11]. Early life exposure is primarily through diet, air, and water during pregnancy and nursing. In the case of lead (Pb), experimental data has revealed that children who were exposed to Pb had altered brain volumes. Early postnatal Pb exposure impairs learning abilities more severely than it does in older animals [12]. Occupational exposure to cadmium (Cd) for a long period of time slows down psychomotor functioning [13]. The hippocampal region of the brain was found to be disrupted in an in vivo investigation with cadmium. Fluoride is known to be excreted in breast milk and can penetrate the placental barrier. It can alter neural networks in various region of the brain, including the cerebellum, motor cortex, and hippocampus, leading to functional deficiencies in memory and learning as well as anxiety-depressive behaviours [14, 15]. Aluminum is the third most abundant metal present on earth. Food, water, industrial waste, pharmaceutics, etc. are all sources of exposure that can lead to significant neurological impairment [16, 17].

Sirtuins are categorised under class III histone deacetylases which are oxidised NAD^+^ dependent enzymes. There are 7 sirtuins out of which sirt 1 has been explored in abundance. Axon extension [18], neurite outgrowth [19], dendritic branching [20], and the cellular destiny of neuronal precursor cells [21, 22] are just a few of the crucial roles that sirtuins play during development. Additionally, these proteins have a significant impact on circadian rhythmicity [23–28], endocrine function [29], and feeding behaviours [30] in the hypothalamus. SIRT1 plays an essential function in neurogenesis, synaptic plasticity, memory formation and neuroprotection [31]. It is a fundamental factor for metabolism of glucose and lipids, DNA damage repair, and transcription via deacetylating transcription factors and histones [32]. By altering p53 and the FOXO family, SIRT1 activation can reduce the generation of ROS [33].

This review writing was based on PubMed based literature search using the following terms in all possible combinations or individually: “potentially toxic trace elements and neurodevelopmental toxicity” (26 results), “SIRT1 and lead (Pb) exposure” (11 results), “SIRT1 and fluoride exposure” (11 results), “SIRT1 and aluminum exposure” (05 results), “SIRT1 and cadmium exposure” (12 results). This PubMed search resulted into a total of 65 published articles in English language and after further scrutinization only 08 original articles, published between Jun 2016 to Feb 2023, with evidence focused only on involvement of SIRT1 in perinatal and neonatal potentially toxic trace elements exposure related neurodevelopmental toxicity in *in-vivo* and similar *in-vitro* studies were selected.

**Table 1:** Summary of clinical evidence for potentially toxic trace elements-based neurotoxicity

S. No.	Element name	Dose and route of exposure	Duration of exposure	Clinical features	Reference
1	Lead	Exposed through maternal blood, environmental exposure (Lead smelting community)	From prenatal to up to seven years of age	Neuropsychological developmental deficit which includes decreased IQ in children.	[34]
2	Arsenic	10 to 50 ppb, in drinking water	1 year to decades	Peripheral neuropathy, reduction in both small myelinated and unmyelinated fibers, CNS impairment.	[35]
3	Aluminum	8.2–9.4 µL/L aluminum dust exposure	30–32 years	Neurological disorders which include mild cognitive impairment.	[36]
4	Mercury	Exposed through maternal blood (Maternal consumption of pilot whale meat)	Prenatal exposure	Cognitive dysfunction in children aged 7 years: poor memory, attention, language, and motor dysfunction.	[37]
5	Cadmium	Exposed through maternal blood (Maternal exposure to environmental cadmium)	Prenatal exposure	Reduction in umbilical cord serum BDNF levels, Lower develop- mental quotients in 12 month old infants.	[38]
6	Fluoride	Exposed through maternal blood (Fluoride intake through diet during pregnancy)	Prenatal exposure	Decreased cognitive outcome in 2 year old male offspring.	[39]

## Overview of SIRT1

Sirtuins are extensively present in both prokaryotes and eukaryotes [40]. An enzymatic domain with roughly 250 amino acids is common among members of this protein family. They were initially recognised as genetic silencing factors because they resembled the silent information regulator (*SIR2*), first discovered in *Saccharomyces cerevisiae* [41]. In mammals, seven sirtuins (SIRT1-SIRT7) are reported and they have been linked with caloric restriction and ageing [42]. Furthermore, they regulate various biological processes at the epigenetic level, including DNA repair, cell metabolism and survival, senescence, and proliferation. They are frequently connected to ageing and diseases of old age as well [43, 44].

The most researched sirtuin member, SIRT1 has numerous functions, including controlling cell cycle and maintaining energy balance [41]. Studies collectively found that SIRT1 has a role in the pathophysiology of neurological conditions, ageing, cancer and metabolic diseases [45]. NAD^+^ dependent mammalian SIRT1 interacts with different substrates in its C- and N-terminal extensions, including p53, FOXOs, NF-κB, PGC-1α, and histones (H3 and H4), to influence a number of cellular processes [46, 47]. Various studies showed that activating SIRT1 can protect neurons from death and degeneration in *in-vivo* models of neurodegenerative diseases [48]. A class of substances known as sirtuin activating compounds (STACs) can improve the effects of sirtuin [49–51]. SIRT activators with neuroprotective effect is summarised in Table 2.

**Table 2:** Summary of various SIRT1 activators with neuroprotective effect

S. No.	SIRT1 activator	Neuroprotection in animals	Mechanism of action	Other benefits	References
1	Resveratrol	Neuroprotection against AD, PD and HD.	1) Upregulates SIRT1 deacetylase activity 2) Activation of AMPK signalling pathway 3) Increase in PGC-1α and NRF-1 mRNA expression	Cardioprotective, anticancer, anti-inflammatory and antioxidant.	[52–56]
2	Quercetin	Neuroprotection in Alzheimer’s disease, improves cognitive disorder in ageing.	1) Upregulates Sirt1 activity 2) Inhibits Aβ synthesis 3) Reduces ROS production	Antioxidant, antiviral, cardioprotective and anticancer.	[57–62]
3	Curcumin	Neuroprotection in Alzheimer’s disease.	1) Upregulates SIRT1 2) Reduces Aβ25-35 toxicity	Anticancer effects, antidiabetic, anti-inflammatory and cardioprotective.	[55, 63, 64]
4	Vitexin	Protection against neurological deficits and neuronal damage.	1) Upregulates SIRT1 activity 2) Activation of MAPK signaling pathway	Cardioprotective, antioxidant and anti-inflammatory.	[65, 66]
5	CoQ10 Precursors (Solanesol)	Neuroprotection in bipolar disorder, AD, PD and HD.	1) Upregulates SIRT1 level 2) Increase in brain antioxidant activity 3) Improves mitochondrial function 4) Restores cholinergic function	Antioxidant and antiageing.	[67–69]
6	Ginseng	Neuroprotection in Cerebral Ischemia.	1) Upregulates SIRT1 activity 2) Inhibits TLR4/MyD88 signalling pathway 3) Inhibits NF-κB transcriptional activity	Antidiabetic, antioxidant and cardioprotective.	[70–72]
**7**	Protocatechuic acid	Improves cognitive function and brain injury, and protection against Parkinson’s disease.	1) Elevates SIRT1 activity 2) Inhibits NLRP3 inflammasome 3) Inhibits NF-κB pathway 4) Reduces oxidative damage	Antioxidant, anticancer effect and anti-inflammatory.	[73–75]
8	Melatonin	Neuroprotection in Alzheimer’s disease and improves cognitive function.	1) Activates SIRT1 2) Inhibits NF-κB pathway 3) Inhibits Aβ and P-tau synthesis 4) Improves mitochondrial function 5) Decreases oxidative stress	Cardioprotective, antioxidant and anti-inflammatory.	[76–80]
9	Catechins	Neuroprotection and improves cognitive deficits in AD.	1) Activates SIRT1 2) Ameliorates neuroinflammation 3) Inhibits Aβ and P-tau synthesis	Antioxidant and can be used against heavy metal poisoning (Metal chelating properties)	[51]

## Physiological and Pathophysiological Role of SIRT1 in Brain Neurodevelopment

The SIRT1 protein is expressed in many of the vital organs, including the brain, heart, kidney and liver. Numerous studies have shown that sirtuins are essential for neurodevelopment [81, 82]. Mammals have 7 known sirtuin enzymes (SIRT1-7) which are highly expressed in certain brain tissue regions [83]. Specific localization of various sirtuins (SIRT1-7) and their functions in the brain is summarised in Table 3. SIRT1 is present throughout the brain, but it is most prominent in the hippocampus, thalamus, and the solitary tract [84, 85]. It is also an important element of many interconnected regulatory pathways, directing the production of axons and dendrites required for neuronal growth and cognitive development, as well as protecting them from stress [84]. SIRT1 also inhibits NF-κB to restore protein homeostasis, increase neuronal plasticity by increasing transcription of key genes involved in cognitive function, reduce ROS production and thus improve mitochondrial function, and suppress persistent chronic inflammation [84].

SIRT1 plays a crucial role in maintaining normal synaptic plasticity and memory [86–89] by controlling the expression of CREB through post-transcriptional regulation, which is mediated by a brain-specific microRNA [90]. SIRT1 normally regulates miR-134 via a repressor complex containing the transcription factor YY1, and unchecked miR-134 expression after SIRT1 deficiency resulted in downregulated expression of CREB and BDNF, impairing synaptic plasticity, including long-term potentiation (LTP) and memory formation [91–93]. SIRT1 regulates processes like oxidative stress, neuronal differentiation, and neurogenesis to maintain the integrity of the brain [90]. They also modulate several gene components such as p53 [94], the FoxO family [95], NF-κB [96], and PGC-1α [97, 98]. The cellular life cycle and energy production are both altered by changes in these gene components as a result of their deacetylation. SIRT1 deacetylates lysine residues by cleaving NAD^+^ into nicotinamide and 10-O-acetyl-ADP-ribose or 20- and 30-O-acetyl-ADP-ribose [99, 100]. SIRT1 can stimulate Akt phosphorylation and activation [101]. Quite a few studies indicate the significant function of SIRT1 and Akt in neuronal survival [102, 103]. SIRT1 deacetylates Akt which in turn promotes its activation [104, 105]. PI3K/Akt/mTOR signalling pathway is essential for axon myelination and oligodendrocyte survival [106]. In the downstream signalling pathway, mTOR functions as substrate of p-Akt and is crucial for the differentiation of OPCs during the course of CNS development.

**Table 3:** Subcellular localization of various sirtuins and its function in the brain

S. No	Sirtuin subtype	Subcellular localization in the brain	Activity	Involvement of sirtuin mediated pathways in neurological disorder	Function	References
1	Sirtuin1	Nucleus and cytosol	Deacetylation	CREB/BDNF pathways, and PGC-1α/NRF1/TFAM pathways	Sustains synaptic plasticity, neurogenesis, mitochondrial function and memory formation.	[44, 63, 107–110]
2	Sirtuin 2	Mainly cytosol and a fraction in nucleus	Deacetylation	RTN4B/BACE1 pathological pathway, and TC-NER pathway	Deacetylates RTN4B and promotes Aβ production and its aggregation in AD, protects neuronal cell death.	[44, 63, 107, 111, 112]
3	Sirtuin 3	Predominantly mitochondria and a fraction in nucleus	Deacetylation and decrotonylation	ADIPOR1/AMPK/PGC-1α signaling pathway in TBI, mitophagy/NLRP3 pathway, and FoxO3a-SOD2 pathway	Maintains mitochondrial homeostasis and antioxidant system, inhibits hippocampal tissue injury & inflammation, and improves cognitive function, promotes mitochondrial complex I activity and attenuates oxidative stress.	[44, 63, 107, 113–115]
4	Sirtuin 4	Mitochondria	Deacetylation and ADP-ribosylation			[44, 63, 107]
5	Sirtuin 5	Predominantly mitochondria and a fraction in cytosol and nucleus	Deacetylation, desuccinylation, demalonylation and deglutarylation	TRK-A and p75NTR signaling pathways	Promotes cell proliferation and survival, facilitates learning and memory, and prevents neuronal inflammation and apoptosis.	[44, 63, 107, 116]
6	Sirtuin 6	Mainly nucleus and a fraction in cytosol	ADP-ribosylation, deacetylation and deacylation	FOXC1/EZH2, and transcription factor YY1	Protects neuroinflammation and brain injury. Maintains brain mitochondrial function and prevents neurodegenerative illnesses.	[44, 63, 107, 117, 118]
7	Sirtuin 7	Nucleus	Deacetylation	IFN*-*γ	Regulation of cell proliferation and reduction in cytokine production,	[44, 63, 107, 119]

## How do Potentially Toxic Trace Elements Enter the Brain?

Neurotoxins are generally absorbed through the skin, lungs or gastrointestinal tract and subsequently circulate throughout the body. By passing through the BBB and CSF, they can enter the brain through the blood and subsequently travel to a specific region of the brain [120]. The BBB significantly restricts the distribution of non-lipophilic compounds in the brain [121]. According to one study, defensive efflux systems such as ATP-binding cassette and P-glycoprotein exist to prevent toxic elements from entering the brain [122]. Despite these defenses, these hazardous substances can enter the brain regions affected by choroid fluxes due to the compromised integrity of the blood-cerebrospinal fluid barrier. Another study discovered that toxins build up in the BBB and CSF before reaching the brain [123].

Researchers have investigated the roles of specific toxic elements in disrupting the BBB to allow entry to the brain. Toxic elements imitate the behaviour of necessary nutrients in order to use physiological ionic transporters to cross the BBB. A study found that in humans, choroid plexus Pb levels were 100 times higher than the brain cortex [124]. Other toxic trace element, such as cadmium can also pass through the BBB in rats [125]. In both developing and adult rats, cadmium readily enters and accumulates [126], and strongly binds to metallothionein (MT-III) [127]. MT-III found in cerebral cortical neurons is a macromolecule that contains sulphur [128].

Neurotoxins are ingested at several stages of life, including the embryo, foetus, newborn, child, adult, and elderly. The quantity of toxic trace elements in the brain may vary greatly between individuals and may highly be dependent on the development of the brain barrier system [129]. Evidence of element transfer in the foetal stage has been discovered by certain experimental research. While cadmium builds up in the placenta during pregnancy, it seems that its transfer to the foetus is limited [130]. Lead concentration in maternal serum is almost similar to that in foetus because it does not accumulate within the placenta [131]. Trace elements cadmium and lead work together synergistically to suppress the expression of the key macromolecule in the BBB, glial fibrillary acidic protein (GFAP) [132]. When cadmium and lead are combined, the BBB function is disrupted, which results in neurological impairments in developing rats. This response is greater than the additive impact on astrocyte toxicity [133].

## Role of SIRT1 in Various Potentially Toxic Trace Elements Associated Neurodevelopmental Toxicity

### SIRT1 as a Potential Target in Neurodevelopmental Toxicity Following Lead (Pb) Exposure in Early life

#### Involvement of SIRT1 in the Prevention of Pb induced Neurodevelopmental Toxicity via Activation of CREB/BDNF Signaling Pathway

Lead (Pb) is an environmental toxin predominantly affecting the developing CNS in both humans and animals, and as per the Agency for Toxic Substances and Disease Registry (ATSDR) − 2017, it is reported as the second most toxic substance [134, 139, 140]. The growing brain is susceptible to Pb toxicity due to immature blood brain barrier leading to accumulation of Pb mainly in the hippocampus thereby resulting in neurodevelopmental toxicity [141, 142]. Epidemiological research has demonstrated a link between early-life lead exposure and cognitive decline, accompanied by anxiety like behaviours [143, 144]. In addition, animal study has reported that Pb exposure in early life might contribute to neurodegenerative disorder like AD in the later life [145, 146]. It is well reported that sirtuin 1 is a key factor in neuronal development and regulates oxidative stress, apoptosis, and autophagy [32, 147]. Wang et al. [134], has investigated the involvement of sirtuin 1 in lead induced hippocampal neurogenesis in rats. In this study, lead exposure throughout the pregnancy until the termination of lactation period (postnatal day (PND) 21) in Sprague Dawley (SD) rats have resulted in increased blood Pb levels in male pups in a dose dependent manner. Furthermore, the Morris water maze (MWM) experiment findings indicated that Pb exposure has led to spatial learning and memory deficits. Likewise, reduction in SIRT1, CREB and BDNF protein levels were observed in the pup’s hippocampus, and mRNA expression results showed the same expression pattern i.e., reduced with Pb exposure. An in vivo model conducted by Feng at el. [108], has reported similar findings that developmental Pb exposure has led to a decrease in SIRT1 and CREB phosphorylation in the hippocampal tissue. The same study also highlighted that administration of resveratrol, a SIRT1 activator, reversed this Pb mediated neurotoxicity. Moreover, the results of Wang et al. [134], revealed that Pb exposure significantly reduced neurogenesis and increased apoptosis in the hippocampus. It was confirmed by reduction in neurogenesis marker, known as Ki-67 and elevated caspase 3 expression in the hippocampal CA1 region, respectively. However, administration of resveratrol (50 mg/kg/d by gavage) showed neuroprotection by SIRT1 upregulation followed by CREB/BDNF signalling pathway activation. These further stimulate neurogenesis and inhibit apoptosis in the hippocampus thereby, ameliorating cognitive impairments in rats caused by early life Pb exposure (Table 4). Considerably, all this data collectively indicates the potential role of sirtuin 1 in Pb induced learning and memory deficits in early life suggesting that sirtuin 1 can be a therapeutic target and its activators can act as an effective intervention to lessen Pb induced neurodevelopmental toxicity.

**Table 4:** Summary of the role of SIRT1 in various potentially toxic trace elements associated neurodevelopmental toxicity

S. No.	Experimental model	Type of species used	SIRT1 modulators used	Outcome	Reference
1	Role of SIRT1 in hippocampal neurogenesis following lead exposure in rats.	Sprague Dawley rats- study conducted in male pups.	Resveratrol – SIRT1 activator (50 mg/kg/d by gavage)	Lead exposure in early life has downregulated the CREB/BDNF signalling pathway and SIRT1 activity thereby, influencing hippocampal apoptosis which caused cognitive deficits in male offspring rats. Resveratrol treatment has shown neuroprotective effect against lead induced toxicity.	[134]
2	Role of SIRT1 in lead induced hippocampal toxicity in rats.	Sprague Dawley rats- study conducted in male pups.	Resveratrol – SIRT1 activator (50 mg/kg/d by intragastric injection)	Lead exposure has downregulated the SIRT1 expression and CREB phosphorylation, thereby causing neurodevelopmental toxicity in male offspring rats. Administration of resveratrol has reversed the neurotoxicity caused by lead.	[108]
3	Role of SIRT1 in lead-induced cognitive dysfunction and brain tissue damage in the developing male rat’s hippocampus.	Sprague Dawley rats- study conducted in male pups.	Monosialoganglioside sodium (GM1) (0.4, 2 and 10 mg/kg by i.p)	Lead exposure has downregulated the SIRT1 expression as well as CREB phosphorylation and BDNF, which further led to apoptosis, neuropathological changes and cognitive dysfunction in the developing male rats. Treatment with GM1 has shown neuroprotective against lead via SIRT1 upregulation.	[109]
4	Role of SIRT1 in developmental Pb exposure induced hippocampal synaptic plasticity deficits.	Sprague Dawley rats- study conducted in male pups.	Resveratrol – SIRT1 activator (50 mg/kg/d by gavage)	Lead exposure has led to developmental synaptic plasticity and memory dysfunction in the male offspring rats via SIRT1 downregulation. However, elevated SIRT1 expression by resveratrol collectively improved the neurotoxic conditions.	[135]
5	Role of SIRT1 in synaptic deficits caused by Pb exposure in both in vitro and in vivo.	Sprague Dawley rats- study conducted in male pups and in vitro PC12 cells.	Resveratrol (50 mg/kg/d by gavage) and SRT1720 – SIRT1 activators.	Lead exposure has resulted in synaptic plasticity and neuronal apoptotic cell damage in the rat hippocampus and PC12 cells respectively. Treatment with resveratrol and SRT1720 has shown neuroprotection against lead via SIRT1 upregulation.	[136]
6	SIRT1-dependent mitochondrial biogenesis against neurodevelopment damage by fluoride.	Sprague-Dawley rats – study conducted in female offspring and SHSY5Y cells.	Resveratrol – SIRT1 activator (200 mg/kg daily by oral gavage) and Nicotinamide (NIC – SIRT1 antagonist) (100 mg/kg daily by oral gavage).	Fluoride induced mitochondrial dysfunction, neuronal death and impaired learning and memory by downregulating SIRT1 deacetylase activity in female offspring rats and SHSY5Y cells. Administration of resveratrol attenuated fluoride toxicity in both rats and SHSY5Y cells.	[110]
**7**	SIRT1 expression in aluminum induced long-term memory deficits in rats.	Wistar rats – study conducted in pups.	Not available	Aluminum exposure has downregulated the SIRT1 expression and impaired learning and memory ability in rat pups, indicating SIRT1 could be a potential target.	[137]
8	SIRT1 expression in cadmium induced mitochondrial dysfunction and oxidative damage in neurons.	Fetal cerebral cortical neurons and PC12 cells.	Not available	Cadmium exposure induced SIRT1 downregulation which further led to mitochondrial damage and oxidative neuronal cell death, which could be responsible for cognitive impairment in animals.	[138]

**Fig. 1:**
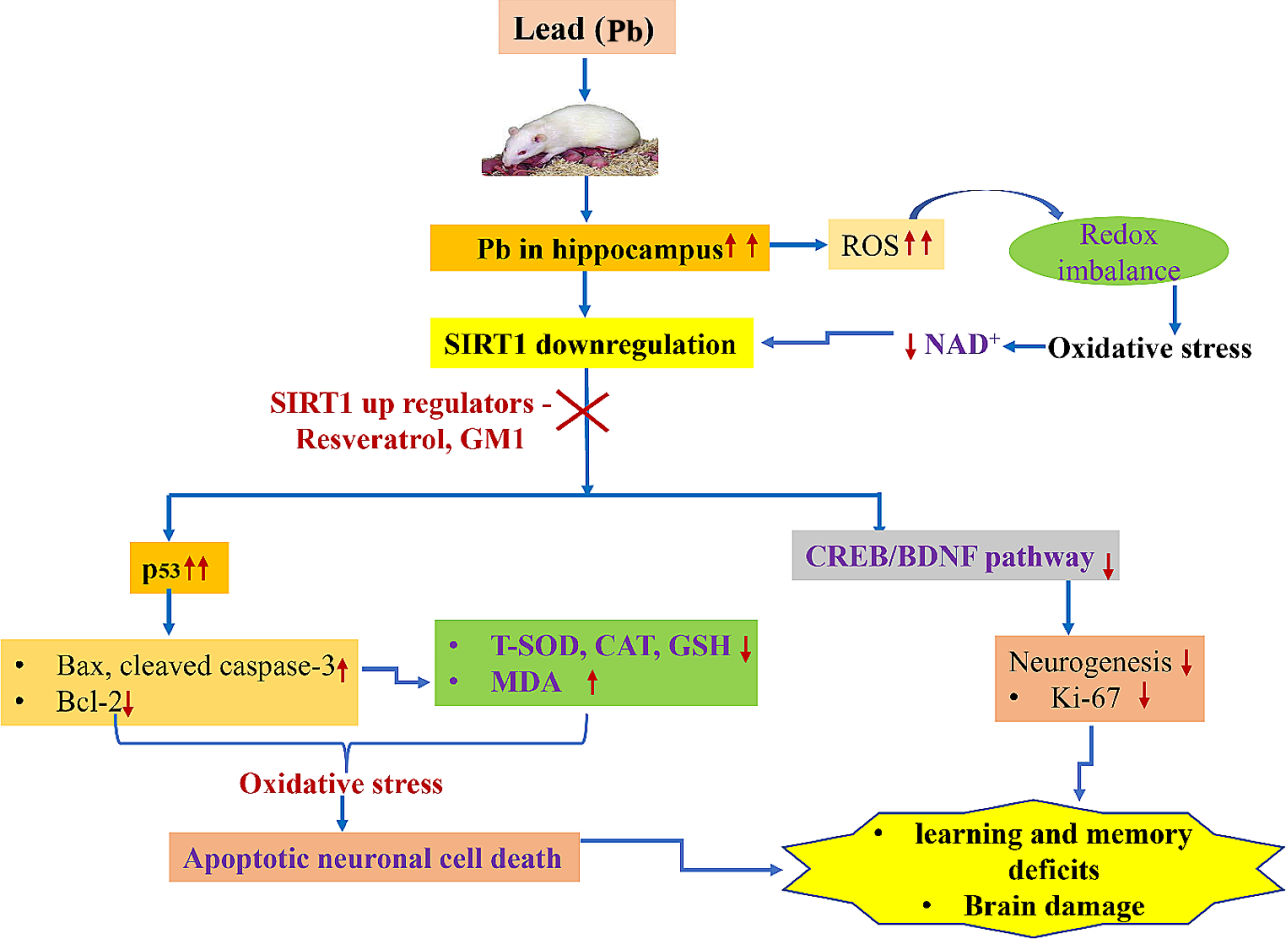
Possible function of SIRT1 in neurodevelopmental toxicity caused by exposure to Pb: Lead exposure in developing rats leads to accumulation of Pb ions in the hippocampus thereby downregulating SIRT1 expression. Pb ions are also linked with overproduction of ROS reducing the NAD^+^ levels and ultimately downregulates the SIRT1 expression. Moreover, SIRT1 downregulation resulted in accumulation of p53 thereby, inducing neuronal cell apoptosis leading to cognitive deficits and brain damage. Due to its deacetylating property, SIRT1 has a pivotal role in attenuating cell apoptosis by deacetylating p53 protein, which is responsible for stimulating neuronal apoptosis by increasing the transcription of Bax and cleaved caspase 3. SIRT1 downregulation has led to decreased CREB and BDNF expressions that primarily leads to reduced neurogenesis and learning and memory deficits. **Note**: This SIRT1 downregulated pathological pathway can be blocked by supplementation with SIRT1 activators like resveratrol and GM1

Likewise, Chen et al. [109], has investigated the possible neuroprotective effect of sirtuin 1 in Pb induced cognitive deficits and brain damage via activation of CREB/BDNF pathway in the hippocampus of developing male rat. It was observed that administration of 0.2% lead acetate has led to an increased serum Pb levels in the hippocampus and resulted in decreased memory and spatial learning capacities. Furthermore, Nissl staining demonstrated that Pb exposure caused notable neuropathological alterations, such as shrinking nuclei, loss of Nissl bodies, and hazy cell borders. This study also confirmed that Pb exposure has induced apoptosis in the hippocampus as marked by reduced antioxidant enzyme activities and elevated MDA levels. In addition, expression of apoptotic proteins by western blot showed the same trend, such as elevated levels of Cleaved Caspase-3 and Bax, and decreased expression of Bcl-2.IHC staining of BDNF in this study has corroborated the western blotting results by Wang et al., that showed BDNF protein decreases with decreased SIRT1 expression due to Pb exposure. However, intraperitoneal administration of monosialoganglioside (GM1) for 10 days starting from postnatal day (PND) 21, upregulated the SIRT1 expression as well as CREB phosphorylation and BDNF in a dose-responsive manner, thereby ameliorating the apoptosis caused by Pb, neuropathological alterations and cognitive deficits in the developing rats. GM1 could be a possible SIRT1 activator which in turn protects against Pb exposure. The sialic acid GM1, which has an oligosaccharide chain and a ceramide unit, is extensively present in vertebrates and predominantly expressed in the CNS [134]. GM1 was reported to exhibit significant neuroprotective effect in various neurogenerative diseases such as AD, PD and HD. Furthermore, supplementation with exogenous GM1 was shown to inhibit apoptosis and oxidative stress [148–152]. Additionally, Chen et al. [108], revealed that Pb exposure reduced the GM1 content in the hippocampus as confirmed by immunofluorescence staining, thereby suggesting that reduction in the GM1 content could be a possible mechanism for SIRT1 downregulation leading to neurotoxicity following Pb exposure in the developing rats. However, due to limited evidence this mechanism needs further exploration. Possible function of SIRT1 in neurodevelopmental toxicity caused by developmental exposure to Pb is summarised in Fig. 1 [108, 109, 134, 153–155].

#### SIRT1 an Essential Regulator of Hippocampal Synaptic Plasticity and Cognitive Function in Lead induced Neurodevelopmental Toxicity

The brain’s ability to retain memory and learning is primarily dependent on synaptic plasticity [156]. A study by Wang et al. [135], has explored the effect of developmental Pb exposure on SIRT1 and synaptic plasticity in the hippocampus of male SD rat pups at PND21. This study revealed that developmental Pb exposure elevated the serum and hippocampal Pb contents which were associated with reduced hippocampal SIRT1 protein and mRNA expressions. It is also well proven that the expression of BDNF, a key regulator of synaptic proteins is mediated by the SIRT1 [157] and similarly, the results of Wang at al., has shown that downregulation of SIRT1 due to Pb exposure has led to decreased hippocampal BDNF protein and mRNA expressions which in turn led to decreased synaptic protein markers which includes presynaptic proteins (Syn-1 and LIMK1) and postsynapting proteins (PSD-95 and NL-1), thereby resulting in spatial learning and memory dysfunction. Interestingly, treatment with 50 mg/kg resveratrol daily upregulated the reduced SIRT1 expression and collectively improved the developmental synaptic plasticity and cognitive impairment in the male SD pups. Another study by Wang et al. [136], was conducted in similar in vivo model as well as in in vitro PC12 cells and reported that reduced hippocampal SIRT1 expression due to Pb exposure is associated with reduction in BDNF expression and other synaptic plasticity related genes such as serine/threonine protein phosphatases 1 (PP1) and Reelin (RELN). However, treatment with resveratrol and SRT1720 (SIRT1 activator) has shown neuroprotective effect via SIRT1 upregulation in both SD pups and PC12 cells. Collectively, these studies suggests that SIRT1 is an essential regulator of hippocampal synaptic plasticity which are closely related to cognitive function and thus SIRT1 can be a potential therapeutic target against Pb induced cognitive impairment in early life.

### Role of SIRT1 in Neurodevelopmental Toxicity Caused by Long Term Fluoride Exposure

Fluoride is ubiquitously present in the environment. As per WHO, the recommended fluoride concentration in drinking water should not be more than 1 mg/L and at this optimal concentration it is beneficial against dental carries and osteoporosis [158, 159]. However, chronic exposure to higher concentrations of fluoride is injurious to health. Ground water is the most common source of fluoride exposure resulting in endemic fluorosis affecting worldwide and most commonly in China, Iraq, and India [160–162]. Apart from dental and skeletal fluorosis, there is ample evidence that researchers are concerned about the detrimental effects of fluoride on neurodevelopment. Various epidemiological studies of neurodevelopmental fluoride toxicity have reported that exposure to high fluoride was responsible for poorer IQ scores in children [163, 164]. Rodent models have shown that developmental fluoride exposure has resulted in learning and memory impairment including brain damage [165–167]. However, there are still no effective therapeutic treatment available for chronic fluorosis.

**Fig. 2:**
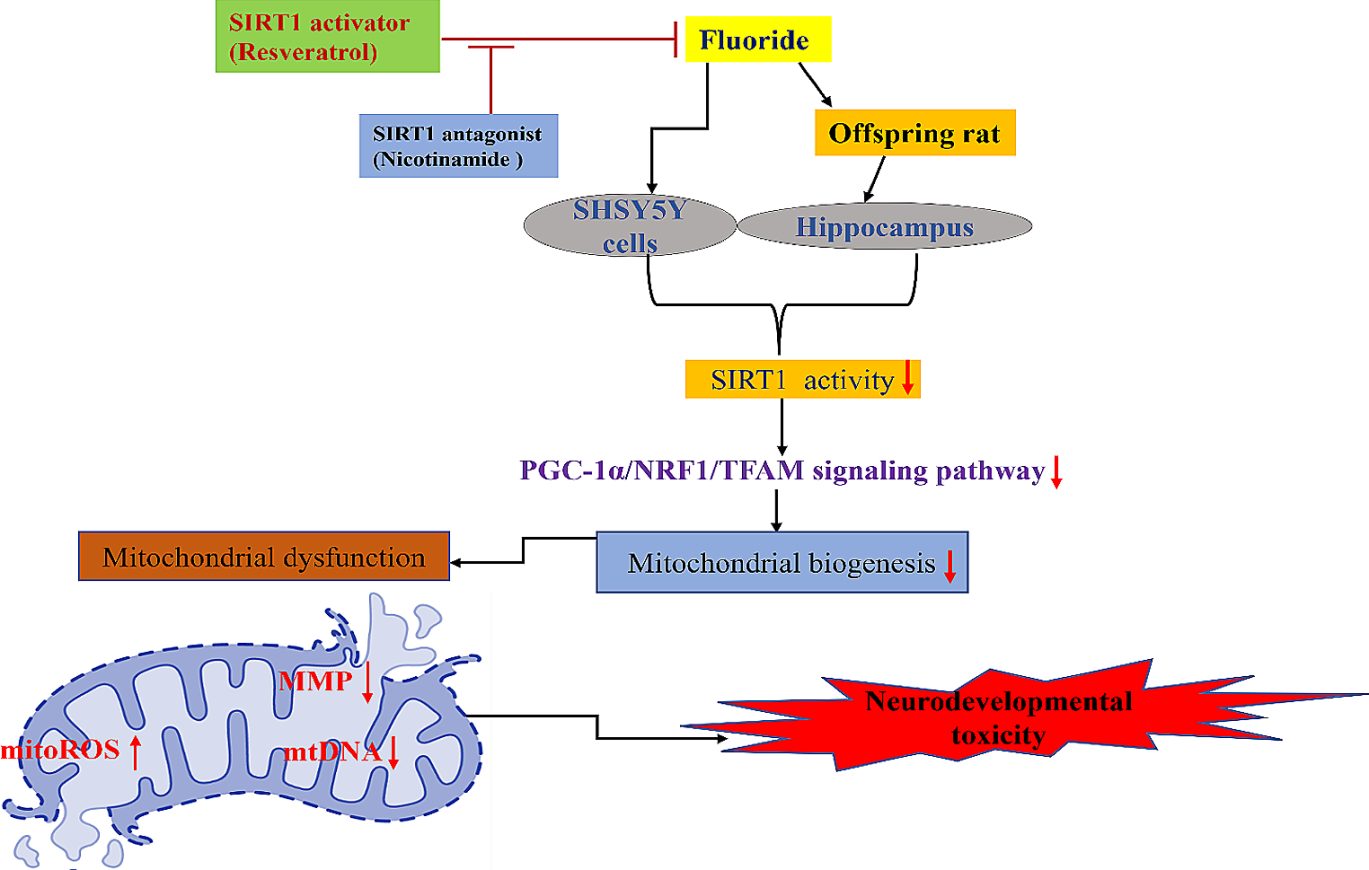
Pathophysiological role of SIRT1 in developmentally fluoride exposure induced neurotoxicity: Developmentally, fluoride exposure leads to accumulation of fluoride in the rat hippocampus which in turn resulted in reduced SIRT1 activity and in SHSY5Y cells. Furthermore, due to reduced SIRT1 activity there is downregulation of PGC-1α/NRF1/TFAM signalling pathway thereby, resulting in decreased mitochondrial biogenesis which further promotes mitochondrial dysfunction and ultimately leads to developmental neurotoxicity in the offspring rats. **NOTE**: SIRT1 downregulated neurodevelopmental toxicity due to fluoride exposure can be reversed by administration of SIRT1 activator such as resveratrol and vice versa with SIRT1 antagonist

Zhao et al. [110], has explored the influence of SIRT1 in fluoride induced developmental neurotoxicity in offspring female SD rats and in vitro SHSY5Y cells. Mitochondrial biogenesis is a key factor which maintains the mitochondrial homeostasis [168, 169]. The results of Zhao et al., revealed that chronic exposure to fluoride induced mitochondrial dysfunction and impaired mitochondrial biogenesis in the offspring female rats and in neuroblastoma SHSY5Y cells, as evident by decreased mitochondrial membrane potential (MMP), mtDNA contents and mtDNA coding gene expressions: CO1, CO2, CO3, ATP6 and ATP8, and with elevated mitoROS production in SHSY5Y cells and CA1 region of hippocampus. In addition, exposure to fluoride significantly lowered the expression of mRNA and protein levels of mitochondrial biogenesis signalling molecules such as PGC-1α, NRF1 and TFAM in both hippocampal tissue and SHSY5Y cells. Earlier studies have demonstrated that SIRT1 regulates mitochondrial biogenesis in neurodevelopment via interaction with signalling molecule PGC-1α [147, 170]. Additionally, SHSY5Y cells infected with adenovirus overexpressing TFAM ameliorated fluoride induced mitochondrial biogenesis impairment as depicted by increased mtDNA contents and its coding gene expressions, thereby indicating that SIRT1 mediates mitochondrial biogenesis via PGC-1α/NRF1/TFAM signalling pathway. Furthermore, fluoride also induced changes in the SIRT1 expression as depicted by decreased SIRT1 deacetylase activity. However, administration of resveratrol attenuated the fluoride induced mitochondrial dysfunction, neuronal death and impaired learning and memory by upregulating SIRT1 deacetylase activity and mitochondrial biogenesis signalling molecules such as PGC-1α, NRF1 and TFAM. Additionally, resveratrol elevated the mtDNA contents and its associated coding gene -ATP6 expressions and also significantly reversed the cognitive impairment induced by long term exposure to fluoride. Moreover, fluoride induced decrease in Nissl bodies and related morphological changes in the developing hippocampal tissue were significantly ameliorated by treatment with resveratrol (Table 4). On the contrary, co-administration with SIRT1 antagonist (nicotinamide) suppressed all the neuroprotective effect of resveratrol in both fluoride exposed offspring rats and SHSY5Y cells (Fig. 2).

### Involvement of SIRT1 in Long Term Memory Impairment Caused by Developmental Aluminum Exposure via CREB/BDNF Pathway

Aluminum is the most commonly found trace element in the earth’s crust [171]. Food, water, antacids, cooking utensils, defense-related enterprises, firearms, and automobiles are all sources of aluminum exposure for humans [172]. Consuming tainted food or water could expose one to hazardous levels of aluminum [172, 173]. Infants are subjected to aluminum through contaminated formula or breast milk [173]. Utilising transferrin-mediated transport 2, aluminum accumulates in distinct brain regions after crossing the BBB [174]. Aluminum exposure is associated with cognitive decline and neurological diseases [173, 175–177].

A study by Yan et al., [137] has explored the function of TORC1 and SIRT1 mediating CREB target gene expression during aluminum-induced deterioration of long-term memory (LTM). Wistar rat pups were exposed to 0.2, 0.4, and 0.6% AlCl_3_ in drinking water during lactation period till postnatal week. The study results indicated that the aluminum content in the blood gradually increased with the increasing doses of AlCl_3_. As the AlCl_3_ dose was raised, the impairment worsened, resulting in fewer cells, a distorted cell layout, and fewer dendrites. The cognitive abilities of rats were considerably affected by AlCl_3_ 40 mg/kg when given daily for 6 months [178, 179]. Additionally, prior research has shown that three months of Al exposure can impair rats’ behaviour in a water maze test [180, 181], which Yan et al., also confirmed in their study. Moreover, few studies have discovered that Ca^2 +^ and cAMP can work together to stimulate TORC1 dephosphorylation, facilitate its nuclear translocation, and ultimately trigger the activation of CRE-downstream target genes [182, 183], which is crucial for memory formation and reactivation. Cognitive capacities could be harmed by SIRT1 deficiency, which is directly linked to neurodegenerative illnesses [184]. According to a prior study, the miR-134 route allowed SIRT1 to modulate CREB’s transcription and expression [185]. miR-134 enhanced transcription levels by binding to CREB mRNA at its 3′UTR region and preventing CREB protein expression in SIRT1 deficiency [185]. In this study, Yan et al. showed that in rats aluminum exerts neurotoxicity by lowering SIRT1 levels (Table 4), weakening the activation of TORC1 and its nuclear translocation, as well as preventing kinase induced CREB phosphorylation. Additionally, CREB’s connection with the BDNF promoter was mediated by SIRT1 [186]. SIRT1 deacetylates methyl-CpG-binding protein 2 to regulate the transcription of BDNF mRNA. In this investigation, treatment with AlCl_3_ showed decreased hippocampal BDNF proteins and mRNA levels as compared to the control rats. Thus, aluminum, via SIRT 1, affects transcription of BDNF gene.

### SIRT1 Activation Attenuates Mitochondrial Dysfunction in the Prevention of Cadmium Induced Neurodevelopmental Toxicity

Cadmium (Cd) is an identified environmental carcinogen and recognised as a neurodevelopmental toxicant [187, 188]. One of the widely dispersed trace elements, cadmium is commonly present in cigarette smoke, drinking water, food, and industrial chemicals. It also has a lengthy biological half-life in contaminated tissues. Cadmium can permeate the BBB and disrupt the nervous system, which can result in neurodegenerative diseases [189]. It has an impact on the foetus and the developing child’s brain in the early phase of development [188, 190]. Cadmium may have an immediate impact on how the CNS develops and is associated with behavioural and cognitive problems in young children [191, 192]. It promotes oxidation and triggers apoptosis in the brain, according to earlier studies [193]. The primary targets of cadmium-induced neurotoxicity are mitochondria.

Long-term exposure to Cd at low concentrations has been proven to induce severe effects on brain metabolism, lowering the levels of norepinephrine, 5-hydroxytryptamine, acetylcholine, and other associated variables, as well as harming the nervous system [194]. Cd is linked to various kinds of neurological illnesses and intellectual disabilities in children, and it may also contribute to memory loss. Serum cadmium level of intellectually handicapped children was shown to be considerably greater than that of able-bodied children, and IQ was found to be adversely connected with serum cadmium levels [195]. In vivo studies have demonstrated that cadmium exposure in young rats can drastically impair learning and memory in adulthood.

A study by Wen et al., [138] has shown that cadmium can induce mitochondrial dysfunction by suppressing SIRT1- mediated oxidative stress [Table 4]. In this study, fetal cerebral cortical neurons and PC12 cells were administered with varying concentrations of cadmium at different time points. Also, the cells were pre-exposed to different doses of Srt1720, which is an agonist of SIRT1. At 18–19 days of gestation, cerebral cortical neurons were extracted from fetal rats. The findings of this investigation demonstrated that cerebral cortical neurons and PC12 cells exposed to Cd had enhanced Mn-SOD activity and overproduced mitochondrial superoxide. Mitochondrial activity is disrupted by oxidative stress, which causes levels of ROS to rise [196]. Previous research has demonstrated that Cd causes mitochondrial dysfunction and the generation of ROS in rat cortical neurons [197]. In rat cortical neurons, Cd increases SOD activity in a concentration dependent way, according to Lopez et al. [197]. The significance of SIRT1 as a target protein for Cd-induced cytotoxicity has become more evident [198, 199]. Additionally, cadmium reduced the survival of neurons by suppressing SIRT1 activity in fetal cerebral cortical neurons and PC12 cells. Srt1720, an activator of SIRT1, was used to treat these cells to study the cadmium-induced neurotoxicity. Srt1720 raised baseline SIRT1 protein levels and prevented cadmium-induced neuronal death. Cadmium generates oxidative damage by downregulating SIRT1 activity, as shown by the inhibition of cadmium-induced overproduction of mitochondrial superoxide and an elevation in Mn-SOD activity after SIRT1 activation by Srt1720. Cadmium exposure decreased the amount of intracellular ATP and MMP in fetal cerebral cortical neurons and PC12 cells but treatment with Srt1720 elevated their levels in these cells. Together, these findings show that Cd causes mitochondrial dysfunction brought on by oxidative stress via repressing SIRT1 expression. Hao et al. [200] discovered that siRNA targeting miR-34a-5p, a suppressor of SIRT1, reduced PC12 cell death caused by Cd exposure. In this work, SIRT1 activation by Srt1720 reduced the overproduction of mitochondrial superoxide caused by Cd and enhanced Mn-SOD activity, demonstrating that Cd caused oxidative damage by suppressing SIRT1 activity.

## Therapeutic Challenges

Numerous studies have been conducted to understand the role of SIRT1 in neurodevelopmental toxicity by targeting SIRT1 in various potentially toxic trace elements induced neurodevelopmental toxicity models. However, the clinical advantages of targeting SIRT1 in various toxic trace elements associated neurodevelopmental toxicity in patients are still missing. Neuroprotective activity of various SIRT1 activators have been reported in rodent models of neurological disorders (Table 2), which could be tested in patients with neurological disorders. However, only resveratrol, a natural SIRT1 activator and few synthetic SIRT1 activators have been utilized clinically to lower the risk in patients with cardiovascular diseases, diabetes, cancer, sleep disorders, ulcerative colitis, atherosclerosis, and AD [201]. It should be noted here that, till date no SIRT1 activators have been tested in patients with neurodevelopmental toxicity due to toxic trace elements exposure in early life. In animal models of potentially toxic trace elements induced neurodevelopmental toxicity, resveratrol has been tested by oral routes and has shown neuroprotection against early life exposure to lead, fluoride, aluminum and cadmium. It is of utmost importance to test the reported SIRT1 activators clinically for prevention and management of toxic trace elements associated neurodevelopmental toxicity in patients and to further develop different dosage forms and safe SIRT1 activators in near future.

## Summary

Exposure to trace elements in early life is a serious concern due to the vulnerability of the developing nervous system to their toxic effects. Of all the seven mammalian sirtuins, SIRT1 is primarily found in all regions of the brain. It plays an important role in maintaining the integrity of the brain by controlling processes like oxidative stress, neuronal differentiation, neurogenesis, and neuronal plasticity. This review summarises the neuroprotective mechanism of SIRT1 in neurodevelopmental toxicity due to potentially toxic trace elements via activation of CREB/BDNF and PGC- 1α/NRF1/TFAM signalling pathways. The available evidence collectively suggests that SIRT1 signalling pathways can be a therapeutic target and its activators can be used as an effective intervention against potentially toxic trace elements (lead, fluoride, aluminum, and cadmium) induced neurodevelopmental toxicity.

## Data Availability

No datasets were generated or analysed during the current study.

## Abbreviations

AD: Alzheimer’s disease
Al: Aluminum
AMPK: Adenosine monophosphate activated protein kinase
ATSDR: Agency for Toxic Substances and Disease Registry
BBB: Blood brain barrier
BDNF: Brain-derived neurotrophic factor
Cd: Cadmium
CREB: Cyclic AMP response element binding protein
CSF: Cerebrospinal fluid
Fl: Fluoride
FOXO: Forkhead O family
GFAP: Glial fibrillary acidic protein
GM1: Monosialoganglioside
HD: Huntington’s disease
mitoROS: Mitochondrial reactive oxygen species
MMP: Mitochondrial membrane potential
mtDNA: Mitochondrial deoxyribonucleic acid
NAD: Nicotinamide adenine dinucleotide
NDDs: Neurodevelopmental disorders
NF-κB: Nuclear factor kappa-light-chain-enhancer of activated B cells
NRF1: Nuclear respiratory factor 1
OPCs: Oligodendrocyte progenitor cells
Pb: Lead
PD: Parkinson’s disease
PGC-1α: Peroxisome proliferator-activated receptor gamma coactivator 1-alpha
PTEs: Potentially toxic trace elements
ROS: Reactive oxygen species
SIRT: Sirtuin
STACs: Sirtuin activating compounds
TBI: Traumatic brain injury
TFAM: Mitochondrial transcription factor A
TORC1: Transducer of regulated CREB activity 1
